# Barriers to implementation of a redesign of information transfer and feedback in acute care: results from a multiple case study

**DOI:** 10.1186/1472-6963-14-149

**Published:** 2014-04-03

**Authors:** Janneke E van Leijen-Zeelenberg, Arno JA van Raak, Inge GP Duimel-Peeters, Mariëlle EAL Kroese, Peter RG Brink, Dirk Ruwaard, Hubertus JM Vrijhoef

**Affiliations:** 1 Department of Health Services Research, School for Public Health and Primary Care (CAPHRI), Maastricht University, Maastricht, The Netherlands; 2 Department of Patient and Care, Maastricht University Medical Center, Maastricht, The Netherlands; 3 Department of General Practice, School for Public Health and Primary Care (CAPHRI), Maastricht University, Maastricht, The Netherlands; 4 Department of Surgery, Maastricht University Medical Center, Maastricht, The Netherlands; 5 Saw Swee Hock School of Public Health, National University of Singapore, Singapore, Singapore; 6 Scientific Center of Care and Welfare (Tranzo), Tilburg University, Tilburg, The Netherlands

**Keywords:** Communication, Redesign, Barriers, Implementation, Acute care, Emergency care, Healthcare providers

## Abstract

**Background:**

Accurate information transfer is an important element of continuity of care and patient safety. Despite the demonstrated urge for improvement of communication in acute care, there is a lack of data on improvements of communication. This study aims to describe the barriers to implementation of a redesign of the existing model for information transfer and feedback.

**Methods:**

A case study with six cases (i.e. acute care chains), using mixed methods was carried out in the Netherlands. The redesign was implemented in one acute care chain while the five other acute care chains served as control groups. Focus group interviews were held with members of the acute care chains and questionnaires were sent to care providers working in the acute care chains.

**Results:**

Respondents reported three sets of barriers for implementation of the model: (a) existing routines for information transfer and feedback in organizations within the acute care chain; (b) barriers related to the implementation method and time period; and (c) the absence of a high ‘sense of urgency’ amongst providers in the acute care chain which would aid in improving the communication process.

**Conclusions:**

This study shows that organizational factors play an important role in the success or failure of redesigning a communication process. Organizational routines can hamper implementation of a redesign if it differs too much from the routines of care providers involved. Besides focussing on provider characteristics in the implementation of a redesigned process, specific attention should be paid to unlearning existing organizational routines.

## Background

Accurate communication is an important feature of seamless care and enhances patient safety [1-4]. Information transfer and patient handovers are noted to be potentially hazardous areas for error in emergency care. Failures in information transfer between healthcare professionals can lead to several errors in care processes, such as poor coordination, inefficient functioning of healthcare providers and longer waiting and throughput times for patients [5,6]. In emergency care, communication failure is known to be the root cause of most adverse events [1,7]. Improving communication in emergency care is therefore necessary, although the subject has received relatively little attention and published studies are of variable quality [1,4,5,7,8]. In the Netherlands, emergency care is partially organised by means of acute care chains. An acute care chain can be defined as the description of the patient flow of a specific diagnosis category, in need of acute care, including agreements on the responsibilities of the healthcare providers involved [9]. Despite efforts of cooperation in a care chain, healthcare providers mention the presence of bottlenecks in communication such as shortage of information on the patients’ case or absence of feedback [9]. A redesign of the communication process, focusing on information transfer and feedback was developed, however implementation of the redesign failed (See Methods: Implementation). The aim of this study was to understand the barriers to implementation of the redesign. The main research question addressed in this article therefore is: what barriers to implementation of redesign of acute care in the Maastricht Heuvelland area are perceived by healthcare providers? On its turn, understanding barriers to implementation helps to create a solid understanding of efforts necessary to improve communication in emergency care. Hence, even in broader perspective, there is a need to prevent under-reporting of research results [10]. In addition, this study also aims to provide information to overcome the existing knowledge gap in improving communication in emergency care.

## Methods

Because of the explorative nature of the research question, a multiple case study comprising six cases, using mixed methods was carried out between October 2009 and April 2011 in the Netherlands. The redesign was implemented in the acute care chain for acute abdominal complaints (AAC) with the five other acute care chains serving as control groups.

### Intervention

**Figure 1:**
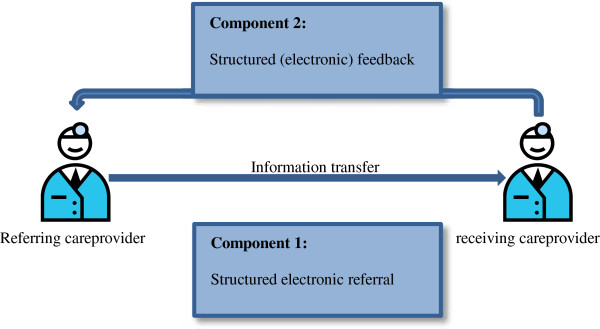
Redesign for information transfer and feedback.

The redesign for information transfer and feedback aimed to improve information transfer in two ways (Figure 1):

1. A standardized electronic referral form to be used between all healthcare providers involved in the acute care chain should ensure availability of the right information for the right healthcare provider, in the right format at the right time.

2. A standardized feedback form to be used between all healthcare providers involved in the acute care chain should help the healthcare providers to continuously improve the quality of information transfer. The healthcare provider can state his or her preferences for feedback at each referral in the acute care chain and feedback can be requested on information transfer, medical performance, the care process and the referral.

Literature on information transfer and feedback [11-14] combined with input from healthcare providers working in acute care chains served as input for the redesign. Three consensus meetings with professionals of the acute care chain for AAC were held in order to develop the redesign. A researcher (JVLZ) chaired these meetings and provided the professionals with adjusted versions of the redesign.

### Implementation

For the implementation of the redesign an implementation group was established. This group consisted of an implementation group leader (PB), a content related coordinator, a logistical coordinator (JVLZ), and five opinion leaders active in the acute care chain for AAC. Being involved at the management level in the trauma center, the implementation group leader was considered to be an opinion leader. The opinion leaders were considered capable to influence the other healthcare professionals working in the acute care chain for AAC, as they were either positioned at an intermediate management level of one of the organizations involved or were considered to be experts on the field of AAC. Implementation activities consisted of the introduction of the redesign to management of the Emergency Care unit and its staff, the ambulance staff, the GPs and staff working at the out-of-hours primary care service (OHPCS). These groups were repeatedly informed of the implementation and use of the redesign, by means of newsletters, emails and presentations. During the implementation period (June 2010 – January 2011), the implementation group held monthly meetings in which goals were set and activities were defined and adjusted based on an intermediate evaluation. The redesign of the information transfer and feedback was hardly implemented, if at all. The first component of the redesign (i.e. the structured electronic referral) was not implemented and the second component of the redesign (i.e. the structured feedback) was implemented but sporadically used. The feedback form was implemented in paper form, and of the 80 forms distributed in the care chain, only four were used. Of these four forms, one was used accurately, one was ripped apart and had to be corrected and replaced and two were not filled in properly.

### Research area

The selection of cases follows from an assignment of the Dutch Ministry of Public Health, Sports and Welfare of the 11 regional trauma centers in which five acute care chains are defined. These being: (a) cerebrovascular accidents (CVA), (b) acute myocardial infarction (AMI), (c) acute hip traumas (AHT), (d) acute psychiatric care (APC) and (e) acute obstetric care (AOC). All of these acute care chains were included in the case study. Located in the Maastricht Heuvelland (MH), other important features of the case study include (1) the existence of a sixth acute care chain – for AAC, (2) the OHPCS located next to the emergency department, (3) in some cases, ambulances from an ambulance base other than Maastricht take care of the transport of patients and (4) the psychiatric hospital located in Maastricht being responsible for hospital care delivery to patients of the MH area with acute psychiatric complaints outside office hours. The OHPCS ‘Maastricht Heuvelland’ is a collaboration of GPs in MH and works during evening, night and weekend shifts [15,16].

### Data collection

Following the implementation period, written questionnaires and focus group (FG) interviews were used to determine perceived barriers to implementation of the redesign. Purposive sampling [17] was used to select respondents for the questionnaires representative for the entire acute care chain. Initially four key categories of healthcare providers were selected; (a) Ambulance caregivers, (b) General Practitioners (GPs), (c) Nurses from the Emergency Department and (d) Medical specialists working at the Emergency Department. These four categories were considered to represent the main disciplines and professionals involved in an acute care chain. The acute care chains for acute psychiatrics, acute myocardial infarction and acute obstetrics, however, are organised in such a way that the Emergency Department is only indirectly involved in these acute care chains. For these acute care chains, the disciplines (a) psychiatry nurse, (b) psychiatrist, (c) nurses from the Cardiac Care Unit, (d) medical specialists working at the Cardiac Care Unit, (e) midwives, (f) nurses from the obstetric unit and (g) medical specialists working at the obstetrics unit respectively were added.

In total, 40 respondents were approached. In the questionnaires respondents were shown 14 characteristics of the acute care chains. For each characteristic, respondents were asked to indicate – on a five-point scale – in what manner they thought the characteristic would influence the quality of information transfer and feedback. Two further questions asked respondents whether they perceived improvement in information transfer and feedback to be important. FG interviews were planned for all acute care chains (size of the groups ranged from three to fourteen participants), with an aim to determine perceived barriers to implementation.

FG interviews were planned during regular meetings of the coordinating group of the acute care chain. A coordinating group consists of representatives of all care providers involved in an acute care chain and were therefore considered to be an optimal representation of the acute care chains. Because we used the coordinating group of the care chains for the FG interviews, we did not balance in age, work experience or hierarchical status of the respondents. Therefore, although participants were considered to be representative for the acute care chains, the composition of the FGs might have caused some bias. Not all acute care chains had such a coordinating group however. In addition, a FG interview was planned with the implementation group. All FG interviews were moderated by a researcher (JVLZ). Before the start of the FG interviews the moderator ensured anonymity and all participants gave their consent.

The FG interviews were semi structured and contained 14 questions for the control care chains and 16 for the acute care chain for AAC. The 14 questions posed to both groups concerned factors possibly influencing implementation of the redesign and were based on implementation literature. More explicit, participants were posed questions about organizational routines [18-20], organizational factors such as care chain coordination and policy of participating institutions [21-23], a sense of urgency for change [21-23] and the implementation methods used. The AAC care chain was posed two extra questions concerning the use of the redesign after implementation and the implementation techniques used. In the control care chains, the moderator presented the redesign as developed for the acute care chain for AAC and asked the respondents to answer as if this redesign were to be implemented into the care chain in which they were involved.

### Data analysis

The responses to the questionnaires were entered into one database for all six acute care chains using SPSS 16.0. Frequency tables were produced for all characteristics of the acute care chains and for each characteristic, a direction of the influence was given (i.e. negative or positive). Missing values were entered into the database as unknown. The FG interviews were recorded and transcribed verbatim to increase validity. Answers were entered into data matrices [17], one for the acute care chain of ACC and one for the control care chains. The rows of the data matrices contained the factors possibly influencing implementation. The ‘bracketing’ technique [24] was used to fill the cells, firstly containing so called thick descriptions (i.e. literal interview passages).Thick descriptions were coded by one researcher – based on the pre-defined concepts – and then transformed into ‘thin’ descriptions – i.e. summarizing the respondents answers per concept. The data matrices containing thick and thin descriptions were discussed by two researchers (JVLZ and AVR) until consensus about the content of the data matrices was reached.

Using two data collection methods allowed us to cross examine the data (data triangulation), increasing validity and reliability as well as objectivity and credibility of the study findings. The RATS guideline was followed to ensure quality of reporting of the study [25].

### Ethical considerations

The study was carried out in accordance with the standards of expected ethical behavior based on The Code of Ethics of the World Medical Association [26]. According to national regulation, full ethical approval was deemed unnecessary because participants in this study were not subject to any acts, nor were they forced to change their behavior at any point during the study [27]. Anonymity was guaranteed to both the respondents of the questionnaires and the FG participants.

## Results

Seven FG interviews were planned with five eventually being held (Table 1). For AOC, no coordinating group existed yet and we were unable to set a date with a representative group of care providers in the field to replace the coordinating group. The coordinating group for AMI was splitting up into two groups at the time of the study and it was therefore not possible to plan a FG interview for this care chain. The length of the FG interviews varied from thirty minutes to one and a half hours, depending on the time available. Time constraints meant not all questions were answered. Questionnaires were sent to 40 care providers and 23 were returned (response rate 57.5%). Response rates varied between acute care chains, varying from 42.8% (CVA) to 80% (APC) (Table 2). Results were categorized according to routines, organization, sense of urgency and implementation methods. Besides barriers to implementation being explored, facilitators to implementation were also discussed by respondents.

**Table 1:** Overview of focus group interviews

**Acute care chain**	**Participants**	**Non response**
CVA	7	
Myocardial infarction	-	Split in care chain coordination, FG interview not feasible
Acute obstetrics	-	No existing FG
Acute hip traumas	11	
Acute psychiatrics	14	
Acute abdominal complaints	4	
Implementation group	3	

**Table 2:** Overview of response to questionnaires

**Acute care chain**	**Questionnaires send**	**Questionnaires returned**	**%**
CVA	7	3	42.8
Myocardial infarction	7	5	71.4
Acute Obstetrics	8	5	62.5
Acute hip traumas	6	3	50.0
Acute psychiatrics	5	4	80.0
Acute abdominal complaints	7	3	42.9
Total	40	23	57.5

### Routines

Respondents indicated that existing routines in the organization might have acted as barriers for implementation in several ways. Firstly, respondents mentioned that routines differed between the organizations involved in the care chain. Respondents in the acute care chain for AAC mentioned that “*giving feedback is more common amongst specialists than it is amongst GPs, because of peer reviews and handovers at shift change”.* Respondents from the acute care chain for AHT answered *“yes”,* when asked to indicate whether work routines in general differ amongst individual providers involved in the acute care chain. In the FG with the acute care chain for CVA, respondents indicated that “*everyone has their own perspective”* and “*on top of that, all hospitals work differently”*. Secondly, respondents mentioned that the redesign differed from the current routines of organizations involved the care chain in that “*the feedback form is not digital yet”* (AAC)*.* Respondents from the acute care chain for AHT mentioned that “*…we have never done this before so we don’t have a routine. Like we said, we mostly work digitally, so a paper form doesn’t actually fit.”* Participants in the acute care chain for CVA mentioned that “*we want to start a certain treatment as soon as possible, so anything that adds logistic throughput or paperwork to the process is a problem”*, indicating that the redesign adds work to the existing routines*.* Finally, participants mentioned that “*it* [providing feedback] *is not an explicit role between GP, specialist and ambulance caregiver…”* and that *“ it is not a habit - apart from the question of whether professionals find it useful - it is just an extra task”* (AAC). In the FG with the acute care chain for AHT, respondents mentioned that “*For some it’s a habit, but for most it’s not a habit to do this* [providing feedback]*”.* In the acute care chain for APC, respondents noted that “*in acute psychiatric care, we always say, 'don’t just provide feedback on paper, but also call'”,* indicating that the redesign does not match with the routine. Results from the questionnaires demonstrate that 21.7% (information transfer) to 30.4% (feedback) of the respondents believe that routines existing information transfer routines negatively influence implementation. Only 13% of the respondents believe that existing information transfer routines and feedback positively influence implementation.

### Organizational aspects

On an organizational level, the absence of a coordinator for the care chains was mentioned as a possible barrier to implementation. Respondents mentioned that “*there is no such thing as a hierarchy in which a protocol can be established, it should be based on equality”* (AAC)*.* Additionally, the absence of a coordinator “*doesn’t have to be a barrier, but in this case it probably was”* (AAC)*.* Respondents in the acute care chain for AHT confirmed the negative influence of the absence of a coordinator with a simple *“yes”* and indicated that *“it does have an adverse effect; nobody tells you what to do”.* The respondents in the acute care chain for CVA also confirmed that “*we don’t have a care chain coordinator”*. The division of responsibilities and authority between providers in the care chain is believed to negatively influence implementation according to 30% of respondents of the questionnaires. Furthermore, inadequate cooperation between providers in the care chain (26.1%) and the nonexistence of a protocol for information transfer on the care chain level (26.1%) is mentioned as negatively influencing implementation (Table 3). Besides barriers, respondents mentioned a facilitator on this topic. The redesign should fit with the organizational policy according to respondents, thus stimulating implementation. When asked whether the redesign does not fit with organizational policy, respondents indicated “*on the contrary, it is really good to do it according to protocol* [information transfer and feedback]” (CVA). Participants state that “*you should not ask whether it fits* [into the organizational policy], *because it should. It is a quality improvement impulse and you do want the quality of the care chain to improve”* (AHT)*.* Respondents in the acute care chain for AAC concurred, “*we all have a quality system in which you think that way”*. In addition to matching the redesign with organizational policy, cooperation between multiple disciplines and organizations was identified as having a fairly positive influence on implementation by 39.1% and 34.8% of respondents respectively (Table 3).

**Table 3:** Influence of care chain characteristics on implementation

**Feature**		**N**	**Influence on implementation (%)**					
			**Very positive**	**Fairly positive**	**Not positive/not negative**	**Fairly negative**	**Very negative**	**Unknown**
*Routines*	*The existing ways of information transfer in the care chain*	23	0.0	13.0	30.5	21.7	0.0	34.8
	*The existing ways of feedback in the care chain*	23	0.0	13.0	17.4	26.1	4.3	39.2
*Organization*	*The existing division of tasks between care providers in this care chain*	23	0.0	26.1	26.1	8.7	0.0	39.1
	*The current division of responsibilities and authority between care providers in this care chain*	23	0.0	8.7	34.8	13.0	0.0	43.5
	*The current division of responsibilities and authority between organizations in this care chain*	23	0.0	8.7	30.4	13.0	0.0	47.9
	*The inadequate cooperation between care providers in this care chain*	23	0.0	8.7	13.0	26.1	0.0	52.2
	*The nonexistence of a protocol for information transfer on care chain level*	23	0.0	4.3	17.4	26.1	0.0	52.2
	*The guiding of information transfer by managers in this care chain*	23	0.0	4.3	30.4	13.0	0.0	52.3
	*The guiding of feedback by managers in this care chain*	23	0.0	4.3	34.8	8.7	0.0	52.2
	*The cooperation with multiple disciplines in the acute care chain*	23	8.7	39.1	13.0	8.7	0.0	30.5
	*The cooperation with multiple organizations in the acute care chain*	23	0.0	34.8	21.7	13.0	0.0	30.5
**Feature**		**N**	**(%)**					
			Very urgent	Fairly urgent	Neutral	Fairly unimportant	Very unimportant	Don’t know
*Sense of urgency*	*How urgent do you experience the need for improvements in information transfer?*	23	8.7	30.4	21.7	8.7	0.0	30.5
	*How urgent do you experience the need for improvements in feedback?*	23	4.3	39.1	21.7	4.3	0.0	30.6

### Sense of urgency

Respondents from all care chains indicated that a sense of urgency for the improvement of information transfer and feedback existed, although this might not have been perceived as very urgent. Participants mentioned that “*it was the main conclusion from the ROAZ* [Dutch acronym for: Regionaal Overleg Acute Zorg, in English: Regional Consultative body for Acute Care] *meeting two years ago, that there is a lack of feedback. We don’t know what happens with a patient so we don’t have any learning points”* (Implementation Group) and that “*a sense of urgency did exist”* (AAC)*.* Furthermore, respondents from the acute care chain for AHT mentioned that “*… regularly, there is a shortage of information for our patients”* and that the problem “*might not be very urgent, but it is very unpleasant of course”.* When discussing the sense of urgency in the FG with the acute care chain for CVA, it is mentioned that *“reading between the lines, I believe we think that improvement is possible and we also think it is needed”.* Finally, respondents stated that “ *It* [the current state of handover communication] *can always be improved”* and “*it* [handover communication,] *can be faster”* (APC)*.*

The need for improvement of information transfer and feedback is indicated to be fairly to very urgent by 39.1% (information transfer) and 43.4% (feedback) of respondents (Table 3).

### Implementation methods

Possible barriers relating to implementation methods were discussed in the FG interviews with the acute care chain for AAC and with the implementation group (IG). Three barriers were mentioned.

Firstly, respondents stated that “*the approach was top down, that might not work with professionals”* (IG) and “*I think that we might question whether we sufficiently introduced the redesign at the professional level”* (IG). Secondly, the timing of the implementation is mentioned as a barrier; “*Maybe the implementation period influenced implementation, it was holiday season at that time”* (AAC)*.* Finally, the features of the redesign itself - mainly concerning not using the electronic capabilities of an organization - were mentioned as a barrier towards implementation. Respondents mentioned that “*above all, the form should be a digital one, it should be a part of your medical file… it is like that because we live in a digital age. If we were still using paper patient records, this form would have been a part of the record”* (IG) and “*if the form is digital, you don’t have the chance to get it returned blank, you simply get a pop-up from the system and have to fill out the form before you can proceed”*(AAC)*.*

The results of the FG interviews and questionnaires mostly correspond with each other, with the exception of the *sense of urgency* feature*.* Respondents from the FG interviews mention this feature as a barrier to implementation, whereas respondents from the questionnaire indicate to experience a fairly to very high sense of urgency.

An overview of the barriers and facilitators to implementation derived from the FG interviews and questionnaire is shown in Tables 3 and 4.

**Table 4:** Barriers and facilitators mentioned in focus groups

**Acute care chain**	**Barrier**	**Facilitator**
Implementation care chain (acute abdominal complaints)	*Routines*	*Routines*
	- Work routines differ between organizations involved in the care chain	
	- Feedback is not provided formally yet	
	- Providing feedback is not a work routine	
	- A non-electronic form differs from current routines, as electronic systems are used	
	- Procedures of information transfer and feedback are absent	
	*Organization*	*Organization*
	- There is no coordination of the acute care chain	- The redesign should fit into the organization’s policy
	*Sense of urgency*	*Sense of urgency*
	- In practice, the sense of urgency might have been very low	- On a higher organizational level, there was a sense of urgency for improvement
	*Implementation methods*	
	- Top down implementation approach	
	- Implementation during holiday season	
	- Features of the redesign itself	
	*Other factors*	*Other factors*
	- Practical experience shows that care providers were not willing to work according to the redesign	- The redesign is desirable
Control care chains* (acute hip traumas, acute psychiatric care, CVA)	*Routines*	*Routines*
	- Work routines differ between districts and organizations involved in the care chain	
	- The redesign differs from the current work routines	
	- Organizations are used to work with digital systems instead of paperwork.	
	*Organization*	*Organization*
	- There is no coordination of the acute care chain	- The redesign should fit into the organization’s policy
	*Sense of urgency*	*Sense of urgency*
	- A need for improvement in information transfer and feedback is experienced, although may not be very urgent	
	*Implementation methods*	
	- Top down implementation approach	
	- Implementation during holiday season	
	- Features of the redesign itself	
	*Other factors*	*Other factors*
	- Willingness to work with the redesign depends on whether it is digital or not. As a paper version, willingness would not be very high	- The ideas behind the redesign are probably desirable

*Acute care chains for Obstetrics and myocardial infarction are missing here since no FG interview was held with those care chains.

## Discussion

In this study, perceived barriers to implementation of a redesign for information transfer and feedback in acute care chains were defined. Based on the responses of the healthcare professionals, these barriers can be grouped into three main categories, relating to: (a) existing routines, (b) implementation method and (c) a low sense of urgency for improvement. As implementation strategies tailored to specific barriers to change seem to be more effective as general strategies [22], the identification of these barriers provide valuable insight for acute care practice and the field of implementation science.

### The role of existing routines in implementation processes

Most barriers to implementation of the redesign mentioned by respondents related to routines, indicating that changing existing routines might play an important role in successful implementation of the redesign. Organizational routines are often described as having a high level of stability, leading to organizational inertia [20]. At the same time, organizational routines are also described as sources of continuous change, as repetition of the same routine by multiple actors leads to a variety of performances [20]. Recent research shows the importance of memory in changing organizational routines [18]. Whereas transactional memory enhances adaptation to changes in organizational routines, declarative memory – building from past experiences – can act as a barrier [18]. Changing an organizational routine is therefore not only a matter of learning a new routine, it also involves unlearning the old [18,28]. Feedback has shown to be an efficient method in learning new (communication) routines [29,30] and was therefore an important component of the redesign for information transfer and feedback. In line with recent research [31], results show however that providers are largely unknown with providing feedback to each other. Providing only an opportunity for feedback on existing routines was not sufficient for changing them. Literature on unlearning suggests that openness to vulnerability, willingness to listen, reflection of feeling and a high tolerance for raised feelings are important qualities for unlearning [32]. Hence, organizations should focus on creating an environment supporting creativity, vulnerability and openness to stimulate unlearning [32]. In addition, respondents mentioned that the redesign deviated too much from the existing routine. More specific, respondents mentioned that the redesign not being integrated into the electronic systems of the organizations was one of the barriers to implementation. Not only because it introduced extra work, but also because the redesign now deviated from the existing routines. These are important insights, as originally feedback was implemented as a tool aiming to change the existing routine. In practice, the actors in the acute care chain felt confronted with a new routine, whilst also having to change the existing routine. If however, the redesign would have been introduced electronically – as a ‘manual’ variation of an existing routine, i.e. the electronic patient record – chances of successful implementation might have been higher. Not only would adaptation to this new routine probably be easier since it stores new learning (using feedback forms) along with the old (using the electronic patient record) [33,34], it also would have been possible to be stricter on the performance of the new routine, by obligating professionals to use the form before they could continue to work in the patient record.

### The role of implementation methods

The top down approach to implementation was indicated as a barrier in this study. This conclusion helps explain why implementation strategies might be more effective if tailored to the different levels within an organization. Hence, this conclusion might also be related to the previous point of changing organizational routines. Seeing routines as sources of continuous change leads to the understanding that change of an organizational routine originates from endogenous factors [20]. Choosing a top down implementation approach however, is based on providing an external stimulus, rather than searching for endogenous factors that would facilitate the same goal.

### The importance of a high sense of urgency

Although respondents indicated a sense of urgency to be present, the level experienced was generally low. A high sense of urgency amongst the users of an intervention is an important factor in successful implementation [23]. Participants should prioritize the implementation process, which should be addressed in implementation projects [23]. Responses from the questionnaires indicate a fairly to very urgent need for improvement. In this area the lack of an overall high sense of urgency was not enough and acted as a barrier to implementation. In addition, establishing a high sense of urgency amongst all healthcare providers involved might be key to successful implementation here [35,36]. From the results of this study, it can be doubted that throughout the acute care chains, the same sense of urgency was present. Communicating with involved healthcare providers about the problems and possibilities of the redesign could already help to establish a sense of urgency.

### Study limitations

The context of this study and the methods used in this study produce some limitations. First, routines are embedded in organizational rules and can differ from one organization to another, highlighting the importance of organizational features. Additionally, context-specific data collection might be perceived as a limitation. However, as a specific context is the object of study, this is inherent to case study research [37]. Nevertheless, case study research is important to develop a nuanced understanding of the real-life context in which theories are applied [38] as was the specific aim of this study. The number of respondents to the questionnaires (n = 23) can be noted as a limitation to this study, however, purposive sampling and cross examination of data was used to address this problem. A relatively large number of respondents answered questions with “don’t know” – ranging from 26.1% to 52.2%. This could be explained by either a fault in the purposive sampling or by the nature of the questions posed. The use of FG interviews for data collection may also be a limitation of the study. It might be possible that we missed some barriers due to the content and structure of the semi structured FG interviews. Some literature suggests the role of the moderator during the FG interviews might disturb the data collection process; i.e. since the moderator determines the agenda of the FG interview, answers are more or less restricted to this agenda [39]. In this study however, although the agenda for the FG interviews was set by the moderator, the moderator let the participants freely elaborate on each topic. The moderator was well trained and only moved on to the next topic when data saturation was reached. Apart from the influence of the moderator, FG interviews are considered to be an appropriate data collection tool when examining complex behaviours and motivations, as was done in this study [39]. The difference in group size between the FGs may have resulted into different group interactions, but the effect this has on the results of the FG interviews is considered to be small [39]. Coding of thick descriptions into thin description by only one researcher might influence validity of the findings, although we used pre-defined concepts for coding to address this problem. Within the implementation literature, numerous factors are known to influence implementation of healthcare innovations [21,22,40] and not all of these were specifically addressed in this study as we were interested in those barriers perceived by healthcare professionals. The findings of this study therefore do not reflect all barriers to implementation. The outcomes remain important, as the barriers mentioned are perceived by the respondents to have been of significant influence in the selected cases of acute care chains.

## Conclusions

In general, these study results show a number of perceived barriers towards implementation. Most of these barriers were related to organizational routines. The study results underline the importance of understanding that, when implementing a redesign of a process, implementation strategies should be tailored to the different actors involved. Additionally, a high sense of urgency might be an important prerequisite for implementation. Future implementation efforts should therefore start off with the establishment of a high sense of urgency amongst involved care providers. Most importantly, in implementation efforts unlearning the existing routine should receive equal attention as learning to use a new routine. Further research is needed to understand which specific routines and contexts are important to address here and how routines are to be unlearned. Hence, the likelihood of success in future efforts to improve communication in acute care will increase as specific attention is paid to unlearning existing communication routines.

## Competing interests

The authors declare that they have no competing interests.

## Authors’ contributions

JVLZ undertook the data collection, analysis and drafted the manuscript. AVR was study lead and helped draft the manuscript. IDP was a member of the project group carrying out the study and helped draft the manuscript. MK contributed to study development and helped draft the manuscript. DR helped draft the manuscript. PB was member of the project group, leader of the implementation group and helped draft the manuscript. HV contributed to study development and helped draft the manuscript. All authors read and approved the final manuscript.

## Pre-publication history

The pre-publication history for this paper can be accessed here:

http://www.biomedcentral.com/1472-6963/14/149/prepub

## References

[B1] Cheung DS Kelly JJ Beach C Berkeley RP Bitterman RA Broida RI Dalsey WC Farley HL Fuller DC Garvey DJ Klauer KM McCullough LB Patterson ES Pham JC Phelan MP Pines JM Schenkel SM Tomolo A Turbiak TW Vozenilek JA Wears RL White ML Improving handoffs in the emergency department Ann Emerg Med 2010 14 171 180 10.1016/j.annemergmed.2009.07.016 19800711

[B2] Donaldson MS Continuity of care: a reconceptualization Med Care Res Rev 2001 14 255 290 10.1177/107755870105800301 11523291

[B3] Haggerty JL Reid RJ Freeman GK Starfield BH Adair CE McKendry R Continuity of care: a multidisciplinary review BMJ 2003 14 1219 1221 10.1136/bmj.327.7425.1219 14630762 PMC274066

[B4] Manser T Foster S Effective handover communication: an overview of research and improvement efforts Best Pract Res Clin Anaesthesiol 2011 14 181 191 21550543 10.1016/j.bpa.2011.02.006

[B5] Beach C Croskerry P Shapiro M Profiles in patient safety: emergency care transitions Acad Emerg Med 2003 14 364 367 10.1111/j.1553-2712.2003.tb01350.x 12670851

[B6] Sandkuhl K Filipe J Cordeiro J Cardoso J Aalst W, Mylopoulos J, Rosemann M, Shaw MJ, Szyperski C Information Logistics in Networked Organizations: Selected Concepts and Application Enterprise Information Systems. Volume 12 2009 Berlin Heidelberg: Springer 43 54

[B7] Patterson PD Pfeiffer AJ Weaver MD Krackhardt D Arnold RM Yealy DM Lave JR Network analysis of team communication in a busy emergency department BMC Health Serv Res 2013 14 109 10.1186/1472-6963-13-109 23521890 PMC3637459

[B8] Creswick N Westbrook JI Braithwaite J Understanding communication networks in the emergency department BMC Health Serv Res 2009 14 247 10.1186/1472-6963-9-247 20043845 PMC2809061

[B9] Traumacentrum Limburg Failure Mode and Effects Analysis CVA, Myocardinfarct, Heuptrauma, Obstetrie, Psychiatrie In Failure Mode and Effects Analysis CVA, Myocardinfarct, Heuptrauma, Obstetrie, Psychiatrie 2009 Maastricht: Traumacentrum Limburg

[B10] Chalmers I Glasziou P Godlee F All trials must be registered and the results published Br Med J 2013 14 f105 10.1136/bmj.f105 23303893

[B11] Jamtvedt G Young JM Kristoffersen DT O’Brien MA Oxman AD Audit and feedback: effects on professional practice and health care outcomes Cochrane Database Syst Rev 2006 14 1 107 CD000259 10.1002/14651858.CD000259.pub2 16625533

[B12] Sargeant J Curran V Allen M Jarvis-Selinger S Ho K Facilitating interpersonal interaction and learning online: linking theory and practice J Contin Educ Health Prof 2006 14 128 136 10.1002/chp.61 16802307

[B13] Sargeant J Mann K Ferrier S Exploring family physicians’ reactions to multisource feedback: perceptions of credibility and usefulness Med Educ 2005 14 497 504 10.1111/j.1365-2929.2005.02124.x 15842684

[B14] Sargeant J Mann K Sinclair D Ferrier S Muirhead P van der Vleuten C Metsemakers J Learning in practice: experiences and perceptions of high-scoring physicians Acad Med 2006 14 655 660 10.1097/01.ACM.0000232422.81299.b7 16799293

[B15] Huisartsenpost Maastricht Heuvelland: Organisatie [http://www.hapmaastricht.nl/nl/organisatie-huisartsenpost/]

[B16] van Uden CJ Giesen PH Metsemakers JF Grol RP Development of out-of-hours primary care by general practitioners (GPs) in The Netherlands: from small-call rotations to large-scale GP cooperatives Fam Med 2006 14 565 569 16944387

[B17] Miles M Huberman M Qualitative Data Analysis: An Expanded Sourcebook 1994 2 Thousand Oaks, CA: Sage

[B18] Miller KD Pentland BT Choi S Dynamics of performing and remembering organizational routines J Manage Stud 2012 14 1536 1558 10.1111/j.1467-6486.2012.01062.x

[B19] Novak L Brooks J Gadd C Anders S Lorenzi N Mediating the intersections of organizational routines during the introduction of a health IT system Eur J Inform Syst 2012 14 552 569 10.1057/ejis.2012.2 PMC3866022 24357898

[B20] Pentland BT Hrem T Hillison D The (N)ever-changing world: stability and change in organizational routines Organ Sci 2011 14 1369 1383 10.1287/orsc.1110.0624

[B21] Grol R Successes and failures in the implementation of evidence-based guidelines for clinical practice Med Care 2001 14 II46 II54 11583121 10.1097/00005650-200108002-00003

[B22] Grol R Grimshaw J From best evidence to best practice: effective implementation of change in patients’ care Lancet 2003 14 1225 1230 10.1016/S0140-6736(03)14546-1 14568747

[B23] Grol RP Bosch MC Hulscher ME Eccles MP Wensing M Planning and studying improvement in patient care: the use of theoretical perspectives Milbank Q 2007 14 93 138 10.1111/j.1468-0009.2007.00478.x 17319808 PMC2690312

[B24] Denzin NK Interpretive Interactionism 1989 Newbury Park, CA: Sage Publications

[B25] Clark J Godlee FJT How to Peer Review a Qualitative Manuscript Peer Review in Health Sciences 2003 2 London: BMJ Books 219 235

[B26] Declaration of helsinki, ethical principals for medical research involving human subjects [http://www.wma.net/en/30publications/10policies/b3/index.html]

[B27] Ministerie van Volksgezondheid, Welzijn en Sport Wet medisch-wetenschappelijk onderzoek met mensen 2014 BWBR0009408. Den Haag

[B28] Akgun AE Byrne JC Lynn GS Keskin H Organizational unlearning as changes in beliefs and routines in organizations J Organ Change Manag 2007 14 794 812 10.1108/09534810710831028

[B29] Smith S Hanson JL Tewksbury LR Christy C Talib NJ Harris MA Beck GL Wolf FM Teaching patient communication skills to medical students: a review of randomized controlled trials Eval Health Prof 2007 14 3 21 10.1177/0163278706297333 17293605

[B30] Hovlid E Bukve O Haug K Aslaksen AB von Plessen C Sustainability of healthcare improvement: what can we learn from learning theory? BMC Health Serv Res 2012 14 235 10.1186/1472-6963-12-235 22863199 PMC3532388

[B31] Hesselink G Vernooij-Dassen M Pijnenborg L Barach P Gademan P Dudzik-Urbaniak E Flink M Orrego C Toccafondi G Johnson JK Schoonhoven L Wollersheim H Organizational culture: an important context for addressing and improving hospital to community patient discharge Med Care 2013 14 90 98 10.1097/MLR.0b013e31827632ec 23132202

[B32] Rushmer R Davies HT Unlearning in health care Qual Saf Health Care 2004 14 2 ii10 ii15 15576685 10.1136/qshc.2003.009506 PMC1765805

[B33] Bouton ME Context, ambiguity, and unlearning: sources of relapse after behavioral extinction Biol Psychiatry 2002 14 976 986 10.1016/S0006-3223(02)01546-9 12437938

[B34] Edmondson AC Bohmer RM Pisano GP Disrupted routines: team learning and new technology implementation in hospitals Admin Sci Quart 2001 14 685 716 10.2307/3094828

[B35] Kotter JP Leading change - Why transformation efforts fail Harv Bus Rev 1995 14 59 67

[B36] Kotter JP A Sense of Urgency 2008 Boston, Massachusetts: Harvard Business Press

[B37] Yin RK Case Study Research: Design and Methods 2009 4 Thousand Oaks: SAGE Inc.

[B38] Flyvbjerg B Five misunderstandings about case-study research Qual Inq 2006 14 219 245 10.1177/1077800405284363

[B39] Morgen DL Focus groups Annu Rev Sociol 1996 14 129 152 10.1146/annurev.soc.22.1.129

[B40] Chaudoir SR Dugan AG Barr CHI Measuring factors affecting implementation of health innovations: a systematic review of structural, organizationals, provider, patient and innovation level measures Implement Sci 2013 14 22 10.1186/1748-5908-8-22 23414420 PMC3598720

